# Design and implementation of a Primary Health Care (PHC) Toolbox for improving the impact of support from Global Development Partners

**DOI:** 10.1186/s44263-024-00046-5

**Published:** 2024-04-04

**Authors:** Dijana Spasenoska, John Grundy, Lundi-Anne Omam, Irtaza Ahmad Chaudhri, Faraz Khalid, Thomas S. O’Connell, Tova Tampe

**Affiliations:** 1 Department of Social Policy, London School of Economics and Political Science, London, UK; 2 College of Public Health, Medical and Veterinary Sciences, James Cook University Australia, Townsville, Australia; 3 Department of Public Health and Primary Care, University of Cambridge, Cambridge, Cambridgeshire UK; 4 World Health Organization Regional Office for the Eastern Mediterranean, Cairo, Egypt; 5 World Health Organization Headquarters, Special Program on Primary Health Care, Geneva, Switzerland; 6 New York University School of Global Public Health, New York, USA

**Keywords:** Primary Health Care, Global Health, Health policy, Global Health Initiatives, Global Development Partners

## Abstract

Primary Health Care (PHC) is the most equitable and cost-effective way to enhance the health of populations and improve health security and is a requirement for achieving universal health coverage (UHC). Vital to advancing the PHC agenda is effective global health partnerships, particularly with Global Health Initiatives (GHIs) which provide financial support for improving population health. Despite progress, GHI support at times remained parallel to rather than embedded in national health strategies. To improve the impact of GHI support, World Health Organization (WHO) member states requested specific guidance to better align GHI support to national health strategies and PHC principles. We present the PHC-GHI Toolbox as a comprehensive set of resources for use by countries to apply the PHC approach to development of plans for securing and optimally utilizing funding received from GHIs, such as Gavi, the Vaccine Alliance; the Global Fund to Fight AIDS, Tuberculosis, and Malaria (GFATM); and the Global Financing Facility (GFF) as well as other donors. The PHC-GHI Toolbox includes a PHC resource database, GHI-specific overviews, a database of health system strengthening (HSS) investments, COVID-19 funding rapid assessment tool, and a focal point database for identifying expert technical assistance. This paper describes the process undertaken for Toolbox development and outlines its potential applications.

## Background

In 2012, 2015, and 2019, at the United Nations (UN) General Assembly, world leaders committed to achieve universal health coverage (UHC), so “all people have access to the health services they need, when and where they need them, without financial hardship” [1]. Further, they acknowledged that health is a basic human right which every person and every community deserve to enjoy to its highest attainable standards [2]. Pragmatically, Primary Health Care (PHC) is the most equitable, effective, and cost-effective way to attain UHC and to achieve the Sustainable Development Goals and related health targets [3, 4]. The principles of PHC, first laid out in the Alma-Ata Declaration in 1978 [5], were reaffirmed 40 years later by world leaders in the 2018 Astana Declaration acknowledging PHC as foundational to the achievement of Health For All embodied in UHC [6].

PHC is a whole-of-society approach to health for ensuring the highest possible level of health and well-being through provision of care as close as feasible to people’s everyday environment [7]. PHC acts on three interrelated and synergistic components: (1) primary care and essential public health functions as the core of integrated health services, (2) multisectoral policy and action, and (3) empowerment of people and communities [8]. The devastating impacts of the COVID-19 pandemic highlighted the lack of strong and resilient health systems. These lessons contributed to the global health discourse on “building back better” by applying a “PHC-focused” health system strengthening (HSS) approach to increase the resiliency of health systems to adequately respond to public health shocks while continuing to make progress in reducing health inequities [9].

Global Health Initiatives (GHIs) are public–private partnerships that often act as financing mechanisms to support strengthening disease-specific control programs and secondarily to strengthen elements of health systems within countries [10]. GHIs have catalyzed significant funding to support gains on health outcomes in many countries. However, challenges remain, including fragmented investments, operating inefficient parallel governance and reporting systems, and distorting health priorities by concentrating resources on narrow disease control efforts while at times neglecting needed co-investments into building up certain critical foundations of the health system needed to sustain disease-specific results [11, 12].

To address requests by countries for guidance on how to use donor funding, especially from GHIs, in a more aligned manner based on national strategies and PHC principles in 2017, the World Health Organization (WHO) worked with GHIs to use experiences to date to better operationalize PHC-focused HSS, which helped shape the development of a *Primary Health Care for Global Health Initiatives (PHC-GHI) Toolbox* [13]. The Toolbox components are designed for Ministries of Health (MoHs) and their partners to use PHC policy and operational guidance to optimize the sustainable impact of GHIs’ support.

The PHC-GHI Toolbox as outlined in this paper models PHC orientation of systems through more analytic, aligned, integrated, and context-specific approaches. The *analytic approach* enables critical questioning of how to reorient systems and has been embedded in the Toolbox as a search option for identifying resources for this purpose. The *aligned approach* is reflected in the overall Toolbox design, which sees GHI overviews linked to PHC approaches, resource database search option based on PHC levers, and analytic questions aligned to recommended interventions in the global operational framework [8]. The *integrated approach* is reflected in the options available in the Toolbox for undertaking thematic analysis by viewing cross-cutting strategic themes across all operational levers and through integration of the components of the Toolbox into a single web-based portal. The *context-specific approach* is reflected in providing the options for planners to develop analytical questions, identify resources, and design case studies based on local conditions while still retaining the option to access global and regional PHC-related resources and case studies for the same purpose.

A first draft of the PHC-GHI Toolbox was developed in 2021 and was deployed by staff from WHO region of Eastern Mediterranean (EMR) for field testing. In this paper, we share the processes undertaken, consultations conducted, and considerations made, to develop and test the Toolbox and map out its potential applications.

## The PHC-GHI Toolbox development process

The initial impetus for the Toolbox [13] was a series of dialogues initiated by one of the authors (T. O.) in 2017–2018 between WHO, Gavi, Global Fund to fight AIDS, Tuberculosis and Malaria (GFATM), Global Financing Facility (GFF), and UHC Partnership staff on assessing challenges and exploring innovative and feasible ways to use the opportunity of GHIs’ support to advance PHC-oriented HSS and ensuring such efforts were coherent with national strategies to attain UHC. Three objectives were identified: First, improving awareness on the part of GHI and disease-specific technical experts of feasible approaches to operationalize PHC concepts; second, ensuring partners and national authorities working on HSS understood the opportunities and limitations of each GHI’s specific grant-making requirements and processes; third, designing a Toolbox whose contents could support achievement of the first two objectives, which would facilitate countries in managing support from multiple GHIs or donors, which could be updated at regular intervals to ensure relevance, and that reduces the effort needed to find PHC-relevant information for those working on obtaining and managing support from GHIs.

Collaborating with countries to effectively use the funding from Gavi, GFATM, GFF, and WHO’s UHC Partnership constitutes a considerable proportion of technical assistance requests to WHO headquarters and regional offices from its member states. It was decided that these four would be the initial focus of the Toolbox, with subsequent editions including more partners as requested by member states.

From 2018 to 2023, the contents of the Toolbox were developed through extensive and iterative consultations with PHC and disease-specific experts from WHO, GHIs, and development partners at the global, regional, and national levels. Table 1 shows the expertise of the consulted participants. The result was formulation of a series of analytic questions relevant to inserting a PHC perspective into each step of a GHI’s grant design, as well as identification of essential and operationally focused PHC global and regional resources. These resources were cross-referenced under each of 14 PHC operational levers (see PHC resource database component, Table 2). Senior leadership and country support teams from each GHI provided feedback on their specific GHI overview and participated in the development of the case studies. These consultations drove several rounds of revision of the PHC-GHI Toolbox, including capturing changes and updates to PHC-related policies and operational guidance produced by Gavi, GFATM, GFF, and the UHC-P [14–17].

**Table 1:** Focus areas of experts providing feedback on the development of the toolbox

	**Health systems strengthening**	**Health financing & governance**	**Health workforce**	**Communicable diseases**	**Immunization**	**Maternal, neonatal, & child health**	**Emergencies**	**Non-communicable diseases**	**Other**^**a**^
**WHO HQ**	12	4	1	2	2	2	3	2	
**WHOEMR RO**	4			6	1				1
**WHO EMR COs**^**b**^	6			4	4		1	1	14
**Total**	**22**	**4**	**1**	**12**	**7**	**2**	**4**	**3**	**15**
	**Development partners**^**c**^	**GHIs**^**d**^							
**Total**	**13**	**10**							

^a^Includes staff within WHO representative offices, clinical epidemiologist, nutritionist, polio eradication officer, communications officer

^b^Somalia, Yemen, Pakistan, Syria, Djibouti, Iran, Morocco, Sudan, Lebanon, and Afghanistan

^c^Development partners (number of people consulted): ILO (2), WFP (1), USAID (3), NORAD (1), UNICEF (4), FCDO (1), World Bank (1)

^d^GHIs (number of people consulted): Gavi (4), Global Fund (4), and Global Financing Facility (4)

**Table 2:** Components of the toolbox

Component	Description	Purpose
PHC resource database	One-hundred twelve analytic questions extensively cross-linked to resource materials providing operational guidance on implementing each of the 14 levers of the PHC operational framework	*Analyze*: Questions were derived from queries on how to apply PHC principles to GHI grant design and implementation *Integrate*: Questions cross-linked to latest research, providing practical operational guidance *Context specific*: Questions link to country-focused guidance For example: “What are feasible areas for GHI catalytic funding that can help improve the quality of the primary health care workforce?” This question links to WHO’s Global Health Workforce Strategy, as well as to country case studies and other guidance on effective practices to strengthen the health workforce
GHI-specific overviews	A series of detailed briefs on each GHI. Each overview describes one GHI’s strategic goals and mandate, structure, types of support available, operations, and core principles. Currently, overviews exist for Gavi, GFATM, GFF, and the Global Health Architecture around UHC using a PHC approach	*Align*: Informs in-country PHC experts in the health ministry and their partners of opportunities and constraints of each GHIs’ support, including grants and provision of commodities *Integrate*: Informs in-country disease-specific experts and their partners of how to apply PHC operational approaches to integrate plans for GHI support into national PHC planning, budgeting, and monitoring processes
Focal point database	Regularly updated and cross-linked list of global and regional experts. One section lists WHO experts on PHC, health technical areas (e.g., financing, logistics, data), and disease-specific strategies (e.g., TB, vaccines) Second section lists each GHI’s regional, country, and content focal persons	*Align*: Permits rapid identification by country teams of relevant PHC and disease-specific technical leads from WHO, partners, and GHIs and allows countries to engage with them to support design and implementation of various types of support available from GHIs *Integrate*: Supports multi-partner and multidisciplinary collaboration on using opportunity of GHI support to discuss optimal coordination of GHI support with in-country health partners
HSS investments by GHIs	A historical database and dashboards of the amounts spent by GHIs on HSS investments, organized by WHO Health System Building blocks	*Analyze*: Compare and contrasts investments from different GHIs to map areas of investments by GHIs *Align*: Facilitates analysis of overlaps, gaps, misalignment to help identify opportunities for harmonization, and cross programmatic efficiencies
COVID-19 funding rapid assessment tool	The COVID-19 funding rapid assessment tool was developed by the PHC Accelerator of the SDG3 GAP	*Align*: Provide data on health financing support from GHIs for pandemic response and health security, which can be factors into national health financing and budgeting processes *Integrate*: Support countries to identify priority areas for linking national PHC efforts into existing or upcoming funding proposals for pandemic response

Regional consultations were conducted with the African, Western Pacific, and Americas regional offices of WHO. There was an open call from WHO headquarters (HQ) to all WHO regional offices, covering a total of 194 member states, to join as collaborators. No selection criteria were provided for inclusion in the work, and it was up to the regional office to determine how best to engage countries for which this work is most relevant (i.e., low- and middle-income countries and low-income countries receiving external funding from GHIs). The WHO EMRO office expressed immediate interest to be a co-developer of the Toolbox, while the other regional offices provided feedback periodically, but no concrete work plan was established to begin activities in the region. Following EMRO’s expression of interest, a total of four online consultations (two in 2021 and two in 2022) were designed and carried out with countries in the region, where WHO country office health systems and program-specific (e.g., tuberculosis, immunization) focal points were invited. The number of participants during each consultation varied, but some of the representatives included Somalia, Yemen, Pakistan, Syria, Djibouti, Iran, Sudan, and Afghanistan. In 2023, an online consultation with the WHO African Region HSS focal points was also conducted. The consultation consists of a brief presentation of each of the components and an open discussion around key questions the developers have asked. Each participant was given the chance to provide feedback. Participants noted the PHC-GHI Toolbox would assist the application of a PHC approach to program planning for both communicable and noncommunicable diseases. Their inputs led to inclusion of additional country and regional-level resources to enable tailoring of PHC-oriented GHI support to national context, as well as examples of the specific uses of the Toolbox [13].

To ensure the PHC-GHI Toolbox captures other contexts and regions that did not participate in its development, guidance on how to adapt the Toolbox to a specific region or country is provided. Throughout the Toolbox, there is guidance on how to adapt it further, for instance, how to select additional national resources and document those or how to develop case studies. An input form is available for users to directly recommend inclusion of additional resources.

The development of the Toolbox is an iterative process allowing for adaptability and practicality. The initial stages of the development focused on knowledge synthesis by generating relevant materials and their optimization based on feedback received. Following its implementation, the components are contextualized by providing guidance for users to capture various national contexts. In the long run, feedback would be implemented annually, except for the resource database and focal point directory which would be updated regularly as new resources become available or as focal points change.

The PHC-GHI Toolbox’s contents align with the 14 levers of the Operational Framework for Primary Health Care [8], and with the Primary Health Care measurement framework [18], to support nationally led progress towards UHC [8]. The aim of the Toolbox is to provide rapid access to practical guidance and useful practices for those working on planning and implementation of GHI investments, to align them with nationally determined PHC priorities and strategies to strengthen health systems. Components of the PHC-GHI Toolbox include a PHC resource database, GHI-specific overviews, a focal point database for identifying PHC and other expert technical assistance, a HSS investment database, and COVID-19 funding rapid assessment tool (Table 2).

Following the extensive consultative processes, the PHC-GHI Toolbox was piloted in Pakistan in February 2022. Pakistan, as a recipient of funding from three GHIs (Gavi, GFATM, GFF) all committed to PHC, was an ideal test case. It is the first country to conduct the Gavi Full Portfolio Planning (FPP) process that seeks improved integration; it is undergoing a Global Fund planning cycle that similarly is committed to strengthening health systems, and it seeks both GFF and World Bank support to reinforce the health sector. During the initial meeting of the in-country mission for the FPP, the PHC-GHI Toolbox was presented by WHO EMRO staff to WHO Pakistan country office, Gavi, and Ministry of Health staff. Limited number of sub-national representatives were familiarized, but it was noted that in future implementation, more efforts should be made to include greater sub-national representation. The national team used the components of the PHC-GHI Toolbox to optimize Gavi’s FPP support by integrating planning and funding for immunization within a broader package of PHC services [19]. This aligned Gavi’s focus on reaching unvaccinated or under-vaccinated children and communities in the context of strengthening PHC for UHC [20]. The team identified specific ways in which as follows: (i) Gavi funding could strengthen PHC as an optimal approach to achieve and sustain immunization objectives (both *what* activities to support and *how* support is designed and provided), (ii) national strategies and operations to implement PHC could more broadly support immunization activities and results, and (iii) how Gavi support might best link to planned GFATM and GFF/World Bank contributions.

To start, a strategic brief for grant design was prepared based on Gavi’s FPP guidelines [21]. Common objectives and outcomes were grouped, and linkages with the 14 PHC levers were identified and adapted to the immunization context. This was used by the country’s PHC and immunization teams to jointly use an aligned approach to the FPP design, analyze feasible priority activities and outputs for a PHC approach to immunization, and explore ways to integrate country-specific PHC guidance to adapt GHIs funding to national context and needs. The country team noted that the analytical questions in the Toolbox provided significant value in strengthening the grant design process.

**Fig. 1:**
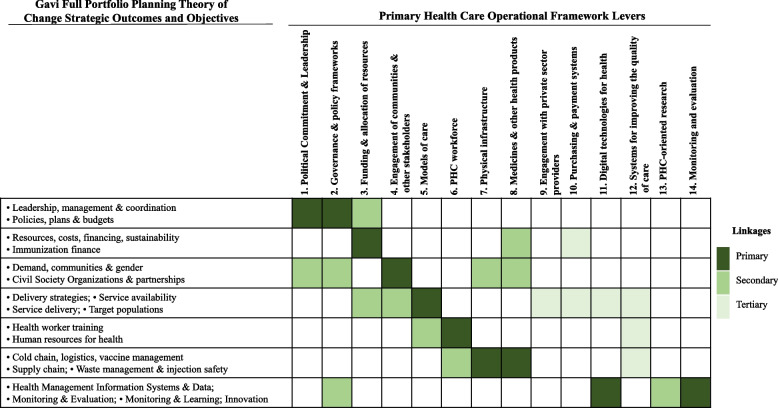
Mapping of Gavi FPP strategic outcomes to PHC levers

Figure 1 illustrates how the analytical questions and resources in the PHC-GHI Toolbox help the user create a link from desired outputs of the Gavi FPP process for the grant making to relevant PHC strategic and operational levers. The type of linkage is indicated by the darkness of the color, where dark green is primary link and light green is tertiary link. For example, for immunization-oriented activities such as optimization of delivery strategies including integrated approaches, the analytical questions help the user consider what is needed to optimize such actions. The primary linkage shows that the user is first directed towards guidance on analyzing models of care. The secondary linkage leads to resources looking at aligning engagement with communities and other stakeholders as well as appropriate allocation of resources the new optimal approach might need. The tertiary linkage then encourages a further discussion looking beyond and considering either other actors such as the private sector or technological advancements through digital technologies for health. All linkages lead to references and case studies detailing practices and options for optimization of service delivery strategies. It is noteworthy that some outputs might be linked to additional PHC levers, but those would be country specific reflective of the activities chosen within the country context.

Applying the PHC perspective to the FPP process was well received, with the team remarking on its potential to strengthen the use of Gavi resources to develop integrated health services with a focus on immunization outcomes. Political leadership at national level, and strong understanding of the PHC concepts at sub-national level, was identified as key enablers.

## Applications of the PHC-GHI Toolbox for PHC orientation of policies, plans, and proposals

The PHC-GHI Toolbox is uniquely designed to support countries to use GHI funding to further enhance PHC orientation of health systems or service activities and minimize fragmented or non-aligned donor-supported activities. There are four immediately practical uses for the PHC-GHI Toolbox:

1. Planning for GHI grant design and implementation by applying PHC principles
2. Applying a PHC approach for the review or revision of a national disease-specific or program-specific health plan, such as national immunization plans, or integration of disease planning processes into the overarching national health planning system.
3. Identifying investment strategies that strengthen country-led stewardship and management of specific investments or service delivery actions in a health proposal or plan supported by one or more GHIs
4. Ensure teams working on the design or revision of various types of GHI support have rapid access to all relevant operational guidance, research, and technical expertise related to PHC.

**Fig. 2:**
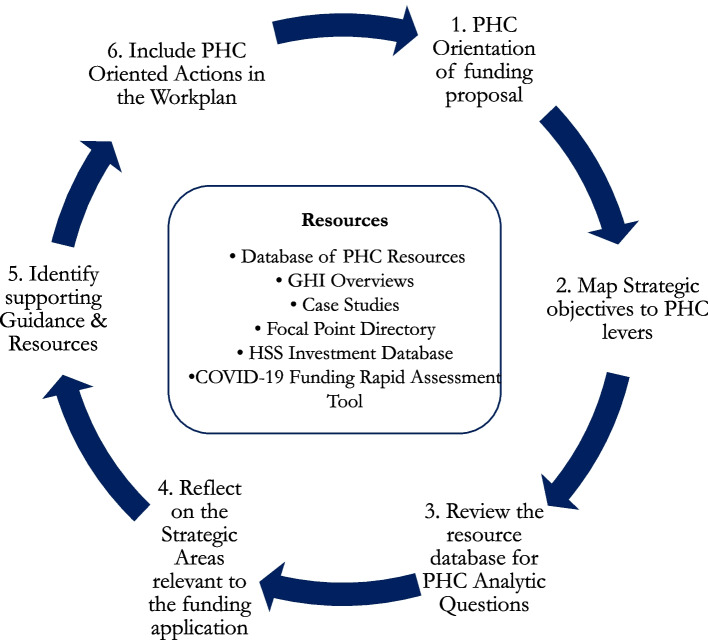
Process steps for using PHC-GHI toolbox to support PHC orientation

The process for using the PHC-GHI Toolbox to support PHC orientation for all use cases is shown in Fig. 2. The first two steps involve catalyzing a nationally led dialogue on contextually relevant ways to map potential GHI-supported activities onto the PHC operational framework levers. This provides national health system planners and their partners to assess which resource and technical gaps should be prioritized for support from GHIs while staying within the allowed mandates and funding requirements unique to each GHI.

The analytic questions available in the Toolbox are aligned to each of the 14 levers of the operational framework, enabling linking of strategic and operational to the PHC approach. Reflection on the strategic areas relevant to the funding application encourages planners to consider synergies across the levers, preventing duplications or misalignments from competing objectives across core health system functions when managing support from multiple GHIs or donors. The PHC-GHI Toolbox also identifies operationally focused resources and case study evidence that provide practical guidance for addressing the questions. While majority of the resources in the database are global resources, under each lever general guidance is provided about relevant resources available at the national level that should be consulted. Examples include documents relating to National Health Strategy, evaluations of financial barriers to access, community health worker policies, etc. There is great scope within the PHC-GHI Toolbox for users to adapt it to local contexts, as well as to identify and add in their own resources and case studies to provide more locally relevant evidence.

This iterative and continuous process completes its cycle through re-evaluating the GHI proposal, plan, or other activity for its consistency with a PHC orientation and modifying it accordingly to best align with national needs, contexts, and priorities. In considering the options for PHC orientation, the main evaluation measures are the extent to which (a) proposed actions relate to some or all of the core PHC principles contained in the relevant WHO and UN General Assembly resolutions on PHC and UHC, (b) proposed activities by GHIs align with national PHC strategic plans and priorities, and (c) the GHI or other health systems funding proposal, plan, or activity aligns with global, regional, and country evidence and guidance on PHC.

## Limitations and considerations

As a set of resources and analytic tools, the aim of the PHC-GHI Toolbox is primarily to provide a more rapid access and much more systematic use of an extensive set of operational guidance for development, implementation, and review of GHI proposals and related activities. It is not primary research but rather a novel way to address the current difficulties country teams have in accessing the needed expertise and guidance to design GHI support within a PHC framework.

The commitment to develop PHC policy and implementation is reliant on the political will to implement and that in turn requires an awareness by health policy makers and planners of value added of a PHC-approach. The PHC-GHI Toolbox is not meant to solve contextual issues such as a lack of political will but instead seeks to enable health planners and their partners to analyze questions and identify resources through a PHC framework. This can then inform country discussions on how to tailor GHI support to better facilitate and accelerate a PHC orientation of donor investments for strengthening elements of health systems, to ensure the results of those investments are fit for purpose now and in the future.

## Conclusions

One of the main challenges in operationalizing the PHC approach has been the lengthy tradition of vertical disease control program management, which, although demonstrating results, has also been critiqued for lack of efficiency, establishment of parallel funding and planning systems, and distortion of national priorities. The PHC approach can improve the effectiveness of health systems through integration of health services and public health functions while also more consistently addressing the social determinants of health through improved community and multi-sector engagement. Reorientation of health systems towards a PHC approach involves a transformation in the way health planners and GHI proposal developers design, implement, monitor, and revise support provided by GHIs. The novel and user-friendly PHC-GHI Toolbox provides a systematic set of resources to assist a country in identifying the evidence required to effectively, efficiently, and sustainably implement PHC orientation of donor-provided resources within an integrative and coherent approach to strengthen health services and health systems.

## Data Availability

The Primary Health Care for Global Health Initiatives Toolbox [13] described in this article is publicly available on the following link: https://extranet.who.int/uhcpartnership/featured/toolbox-primary-health-care-resources-global-health-initiatives.

## Abbreviations

EMRO: Eastern Mediterranean Regional Office
FCDO: The Foreign, Commonwealth & Development Office
Gavi FPP: Gavi Full Portfolio Planning
GFATM: The Global Fund to Fight AIDS, Tuberculosis and Malaria
GFF: Global Financing Facility
GHIs: Global Health Initiatives
HSS: Health system strengthening
HQ: Headquarters
ILO: International Labour Organization
MoH: Ministry of Health
NORAD: Norwegian Agency for Development Cooperation
PHC: Primary Health Care
SDG3 GAP: Sustainable Development Goal 3 Global Action Plan for Healthy Lives and Well-being for All
UHC: Universal health coverage
UN: United Nations
UNICEF: United Nations Children’s Fund
USAID: United States Agency for International Development
WFP: World Food Programme
WHO: World Health Organization
